# Antimicrobial Resistance in *Campylobacter*

**DOI:** 10.3201/eid1007.040580

**Published:** 2004-07

**Authors:** Nicole M. Iovine, Martin J. Blaser

**Affiliations:** * New York University School of Medicine, New York, New York, USA;; † New York Harbor Department of Veterans Affairs Medical Center, New York, New York, USA

**Keywords:** Antimicrobial resistance, Campylobacter, letter

**To the Editor:** We wish to rectify several errors in our commentary, Antibiotics in Animal Feed and Spread of Resistant *Campylobacter* from Poultry to Humans (1). The fluoroquinolone enrofloxacin was approved in 1996 for therapeutic use by addition to drinking water upon the decision of a licensed veterinarian "for the control of mortality in chickens associated with *Escherichia coli* organisms and control of mortality in turkeys associated with *E. coli* and *Pasteurella multocida* organisms" (2). This therapeutic use was withdrawn (3) but is now under appeal. Initial approval and subsequent efforts to withdraw use of enrofloxacin in the United States parallel the earlier trend in Europe and specifically Denmark, where the use of antimicrobial agents as growth promoters has been banned (4).

Enrofloxacin is not approved for prophylactic or growth promotion use in poultry feed as stated in our commentary and in the first section of the flowchart (1). However, when enrofloxacin is added to the drinking water of poultry, large numbers of both ill and healthy animals are exposed to the agent (5). Although extra-label use of enrofloxacin is prohibited, microbiologic culture of either of the cited bacteria is not required before administration (2). Despite the restrictions on enrofloxacin use, emergence of fluoroquinolone-resistant *Campylobacter* species, with poultry as an important source, has been documented in the United States (5,6). Thus the decision to withdraw therapeutic use of enrofloxacin (3) was warranted. Therefore, our conclusion remains: use of enrofloxacin in poultry materially contributed to increase in human infection by fluoroquinolone-resistant *Campylobacter* species. Given the above, our commentary should have been entitled Use of Antibiotics in the Poultry Industry and Spread of Resistant *Campylobacter* to Humans. We regret the errors and hope we have clarified this issue.
